# *SVEP1* as a Genetic Modifier of *TEK*-Related Primary Congenital Glaucoma

**DOI:** 10.1167/iovs.61.12.6

**Published:** 2020-10-07

**Authors:** Terri L. Young, Kristina N. Whisenhunt, Jing Jin, Sarah M. LaMartina, Sean M. Martin, Tomokazu Souma, Vachiranee Limviphuvadh, Fatemeh Suri, Emmanuelle Souzeau, Xue Zhang, Yongwook Dan, Evie Anagnos, Susana Carmona, Nicole M. Jody, Nickie Stangel, Emily C. Higuchi, Samuel J. Huang, Owen M. Siggs, Maria José Simões, Brendan M. Lawson, Jacob S. Martin, Elahe Elahi, Mehrnaz Narooie-Nejad, Behzad Fallahi Motlagh, Susan E. Quaggin, Heather D. Potter, Eduardo D. Silva, Jamie E. Craig, Conceição Egas, Reza Maroofian, Sebastian Maurer-Stroh, Yasmin S. Bradfield, Stuart W. Tompson

**Affiliations:** 1 Department of Ophthalmology and Visual Sciences, University of Wisconsin-Madison, Madison, Wisconsin, United States; 2 Feinberg Cardiovascular Research Institute and Division of Nephrology/Hypertension, Northwestern University Feinberg School of Medicine, Chicago, Illinois, United States; 3 Bioinformatics Institute (BII), Agency for Science Technology and Research (A*STAR), Singapore; 4 Innovations in Food & Chemical Safety Programme (IFCS), A*STAR, Singapore; 5 Ophthalmic Research Center, Shahid Beheshti University of Medical Sciences, Tehran, Iran; 6 Department of Ophthalmology, Flinders University, Flinders Medical Centre, Adelaide, South Australia, Australia; 7 Biocant, Transfer Technology Association, Cantanhede, Portugal; 8 Faculty of Medicine, University of Coimbra, Coimbra, Portugal; 9 School of Biology, University College of Science, University of Tehran, Tehran, Iran; 10 Genetics of Non-communicable Disease Research Center, Zahedan University of Medical Science, Zahedan, Iran; 11 Nikukari Eye Hospital, Tabriz University of Medical Sciences, Tabriz, Iran; 12 Faculty of Medicine, Institute for Biomedical Imaging and Life Sciences, University of Coimbra, Coimbra, Portugal; 13 Center for Neuroscience and Cell Biology, University of Coimbra, Coimbra, Portugal; 14 Genetics Research Center, Molecular and Clinical Sciences Institute, St George's, University of London, Cranmer Terrace, London, United Kingdom; 15 Department of Biological Sciences, National University of Singapore (NUS), Singapore

**Keywords:** glaucoma, modifier, TEK, SVEP1, Schlemm's canal

## Abstract

**Purpose:**

Affecting children by age 3, primary congenital glaucoma (PCG) can cause debilitating vision loss by the developmental impairment of aqueous drainage resulting in high intraocular pressure (IOP), globe enlargement, and optic neuropathy. TEK haploinsufficiency accounts for 5% of PCG in diverse populations, with low penetrance explained by variable dysgenesis of Schlemm's canal (SC) in mice. We report eight families with *TEK*-related PCG, and provide evidence for *SVEP1* as a disease modifier in family 8 with a higher penetrance and severity.

**Methods:**

Exome sequencing identified coding/splice site variants with an allele frequency less than 0.0001 (gnomAD). *TEK* variant effects were assayed in construct-transfected HEK293 cells via detection of autophosphorylated (active) TEK protein. An enucleated eye from an affected member of family 8 was examined via histology. SVEP1 expression in developing outflow tissues was detected by immunofluorescent staining of 7-day mouse anterior segments. SVEP1 stimulation of *TEK* expression in human umbilical vascular endothelial cells (HUVECs) was measured by TaqMan quantitative PCR.

**Results:**

Heterozygous *TEK* loss-of-function alleles were identified in eight PCG families, with parent–child disease transmission observed in two pedigrees. Family 8 exhibited greater disease penetrance and severity, histology revealed absence of SC in one eye, and *SVEP1*:p.R997C was identified in four of the five affected individuals. During SC development, SVEP1 is secreted by surrounding tissues. *SVEP1*:p.R997C abrogates stimulation of *TEK* expression by HUVECs.

**Conclusions:**

We provide further evidence for PCG caused by TEK haploinsufficiency, affirm autosomal dominant inheritance in two pedigrees, and propose *SVEP1* as a modifier of *TEK* expression during SC development, affecting disease penetrance and severity.

Affecting children within the first 3 years of life, primary congenital glaucoma (PCG, MIM: 231300) is potentially a debilitating ocular disease with irreversible blindness.^1^^,^^2^ Elevated intraocular pressure (IOP) is the primary factor for disease progression, causing multiple ocular structural changes, such as globe enlargement (buphthalmos), corneal stretching with thinning and edema, optic nerve damage with characteristic cupping of the optic disc, and retinal ganglion cell loss with concordant patterns of visual field loss and blindness.^2^

In a healthy eye, the IOP is maintained via balanced aqueous humor production in the ciliary body and drainage through the conventional and uveoscleral outflow pathways.^3^^,^^4^ In humans, approximately 80% of aqueous humor drains through the conventional egress pathway, which is comprised of the trabecular meshwork (TM) and Schlemm's canal (SC), a unique hybrid vessel with both vascular and lymphatic properties.^5^^,^^6^ PCG can result from developmental defects in the conventional outflow pathway, leading to aqueous retention and subsequently increased IOP. However, the underlying molecular cause of PCG remains largely undiscovered, with deleterious variants in *CYP1B1*, *LTBP2*, *TEK*, and *ANGPT1* accounting for only 26% of affected individuals from diverse populations.^7^^–^^14^

Heterozygous loss-of-function (LoF) variants in the tunica interna endothelial cell kinase gene, *TEK* (or *TIE2*, MIM 600221), have been estimated to cause 5% of PCG in diverse populations.^13^ The encoded cell membrane receptor is expressed by vascular endothelial cells (ECs) and its phosphorylation signaling is important for maintenance of vascular homeostasis.^15^^–^^19^ High expression of *Tek* has also been detected in the ECs of SC.^6^^,^^20^ In mice, homozygous deletion of the *Tek* gene after heart formation (E16.5) resulted in the developmental loss of SC, highly elevated IOP, globe enlargement, optic nerve damage, retinal ganglion cell degeneration and vision loss.^21^ Furthermore, mice hemizygous for *Tek* develop a variably hypomorphic SC with convolutions and focal narrowings, a hypoplastic TM, and an average 25% increase in IOP compared with control animals.^13^ Importantly, the variable IOP levels were correlated with the degree of SC hypomorphism, thus providing an explanation for the decreased penetrance and variable expressivity observed in human families.^13^

We describe eight PCG families that harbor unreported heterozygous LoF variants in *TEK*, including two nonconsanguineous families demonstrating direct parent-to-child disease transmission over multiple generations. These findings strongly support an autosomal dominant mode of inheritance in these instances. In one pedigree, four of the five affected individuals harbored an additional rare variant in the *SVEP1* (Sushi, Von Willebrand factor type A, epidermal growth factor, and pentraxin domain containing-1) gene (MIM 611691), which we hypothesized may act as a modifier of *TEK* expression, leading to a further decrease in TEK signaling, and subsequent increased disease penetrance and severity.

## Methods

Detailed materials and methods are given in the Supplementary Materials.

### Study Participants

Studies involving human participants were approved by the Ethical Committee of the Faculty of Medicine, University of Coimbra, Portugal; the Ethical Committee of the Shahid Beheshti University of Medical Sciences, Iran; the Southern Adelaide Clinical Human Research Ethics Committee, Australia; the National Institutes of Health Institutional Review Board, Bethesda, Maryland; or the Health Sciences Institutional Review Board at the University of Wisconsin–Madison, Wisconsin. After an explanation of the nature and possible consequences of the study, consent was obtained, and individuals were enrolled.

Patients with PCG were referred by their pediatric ophthalmologist. A clinical diagnosis was confirmed by age of onset less than 3 years, increased IOP (>21 mm Hg), abnormal corneal appearance (diameter >10.5 mm, presence of edema and/or Haab striae), and cupping of the optic nerve head (>0.3 cup to disc ratio).

### Exome Sequencing and Variant Filtering

Exome sequencing was performed at independent institutions as detailed in the Supplementary Materials. Rare variants within exonic and splice site regions were evaluated for likely pathogenicity, including an assessment of evolutionary conservation of the affected residue, *in silico* predictions of pathogenicity, protein stability, and splice site variation, as detailed in the Supplementary Materials. Candidate pathogenic variants were confirmed in the original DNA samples by Sanger sequencing (see Supplementary Table S1 for primers), and segregation analysis was performed whenever familial DNA was available.

### Exon Trapping: Functional Assessment of Splice Site Variation

Exon trapping was performed as previously described.^13^^,^^22^ A 775 bp genomic fragment containing *TEK* exon 11 was PCR amplified from the heterozygous proband of family 7. Amplicons with and without the c.1624+5G>A variant were sub-cloned into the pSPL3 exon trapping vector (Invitrogen Life Technologies, Carlsbad, CA [discontinued]) using XhoI and NdeI endonucleases (Life Technologies).

### Functional Assessment of TEK Missense Variants

*TEK* missense variants were functionally assessed as previously described.^13^ Plasmids encoding *TEK* variants p.G136V, p.V188G, p.Y193C, p.P244R, p.A841V, and p.G1035R were created using site-directed mutagenesis. A plasmid encoding *TEK* with a previously reported nonfunctional kinase domain variant (p.D982A, kinase dead [KD]) was also used.^13^

### Human Ocular Pathology

Enucleated globes were received in 10% neutral buffered formalin and transferred to fresh neutral buffered formalin for 48 hours before opening the eyes. Superior and inferior calottes were removed and the remaining center sections placed in tissue cassettes. Samples were processed through an alcohol series and embedded in paraffin blocks. Sagittal sections were cut, and select slides manually stained with hematoxylin and eosin. All slides were imaged using an Aperio ScanScope CS pathology slide scanner (Leica Biosystems Inc., Buffalo Grove, IL) and Aperio ImageScope software.

### Localization of SVEP1 to Developing Mouse Ocular Outflow Tissues

Mouse tissues were acquired following protocols that were ethically reviewed and approved by the University of Wisconsin–Madison School of Medicine and Public Health Institutional Animal Care and Use Committee, and adhered to the ARVO Statement for the Use of Animals in Ophthalmic and Vision Research. Anterior segments from 1-week-old wild-type (WT) mouse eyes were flat-mount immunostained for SVEP1 and CD31 as detailed in the Supplementary Materials. Briefly, enucleated eyes were immersion fixed in 2% PFA, anterior eye cups dissected, and tissues immunostained using rat anti-mouse CD31 antibody (Cat # 553370, BD Pharmingen, San Jose, CA), rabbit anti-human SVEP1 antibody (Cat # PA5-54436, Thermo Fisher Scientific, Waltham, MA), goat anti-rat IgG Alexa Fluor 594 (Cat # A-11007, Thermo Fisher Scientific), and goat anti-rabbit IgG Alexa Fluor 488 (Cat # ab150081, Abcam, Cambridge, UK) secondary antibodies. Control tissues were also processed without the addition of primary antibodies. Eye cups were washed, flattened on glass coverslips, and mounted with Shandon Immu-Mount (Thermo Fisher Scientific). SC imaging was performed using a Nikon A1RS confocal microscope.

The specificity of the anti-SVEP1 antibody was also verified via immunohistochemical staining sections of human placenta (Supplementary Fig. S3).

### Functional Assessment of *SVEP1* Missense Variant

The *SVEP1*:p.R997C variant was functionally assessed as detailed in the Supplementary Materials. Briefly, *Svep1* cDNA was amplified from mouse placenta and cloned into the pSF-CMV-NEO-COOH-3XFLAG vector (Sigma-Aldrich Corp., St. Louis, MO). A *SVEP1:*p.R997C-expressing construct was generated by site-directed mutagenesis. Constructs were transfected into HEK293T cells (Thermo Fisher Scientific) and SVEP1 detected by immunoblotting with anti-FLAG M2 antibody (anti-GAPDH for loading control). Secreted SVEP1 was detected in serum-free media after 2 days of culture. Cell localization was studied 24 hours after transfection on glass cover slips using an anti-FLAG-FITC antibody (1:500; M2, F4049, Sigma-Aldrich Corp.), ProLong Diamond antifade mountant with DAPI (Thermo Fisher Scientific), and an Olympus BX51 fluorescence microscope.

The ability of extracellular SVEP1 to stimulate *TEK* gene expression in human umbilical vascular endothelial cells (HUVECs) (PCS-100-013; ATCC, Manassas, VA) was assessed as follows: WT-SVEP1, p.R997C-SVEP1, and empty FLAG vector were expressed by HEK293T cells in vascular cell basal medium (VCBM; PCS-100-030; ATCC) supplemented with Endothelial Cell Growth Kit-VEGF (PCS-100-041; ATCC) for 2 days. Cell-free conditioned media was collected, added to growing HUVECs for 24 hours, the cells lysed and processed using a Cells-to-cDNA II kit (Thermo Fisher Scientific), and quantitative real-time PCR performed to assess *TEK* gene expression (TaqMan probe Hs00176096_m1, FAM-MGB; Thermo Fisher Scientific). Human *ACTB* endogenous control (FAM/MGB probe, non-primer limited; Thermo Fisher Scientific) was used for RNA normalization. Three independent experiments were performed totaling 10 biological samples for each condition, and four technical replicates averaged for each sample. *TEK* expression in cells treated with WT- or p.R997C-SVEP1 was calculated as fold change relative to untreated (empty FLAG vector) cells using the 2^−ΔΔCt^ method.^23^ Statistical significance between the means of condition pairs were assessed using the two-tailed *t* test. The standard deviation of each condition was corrected to account for the three experiment means used to pool the data.

## Results

### Rare *TEK* Variants Identified in Eight PCG Families

**Figure 1.**
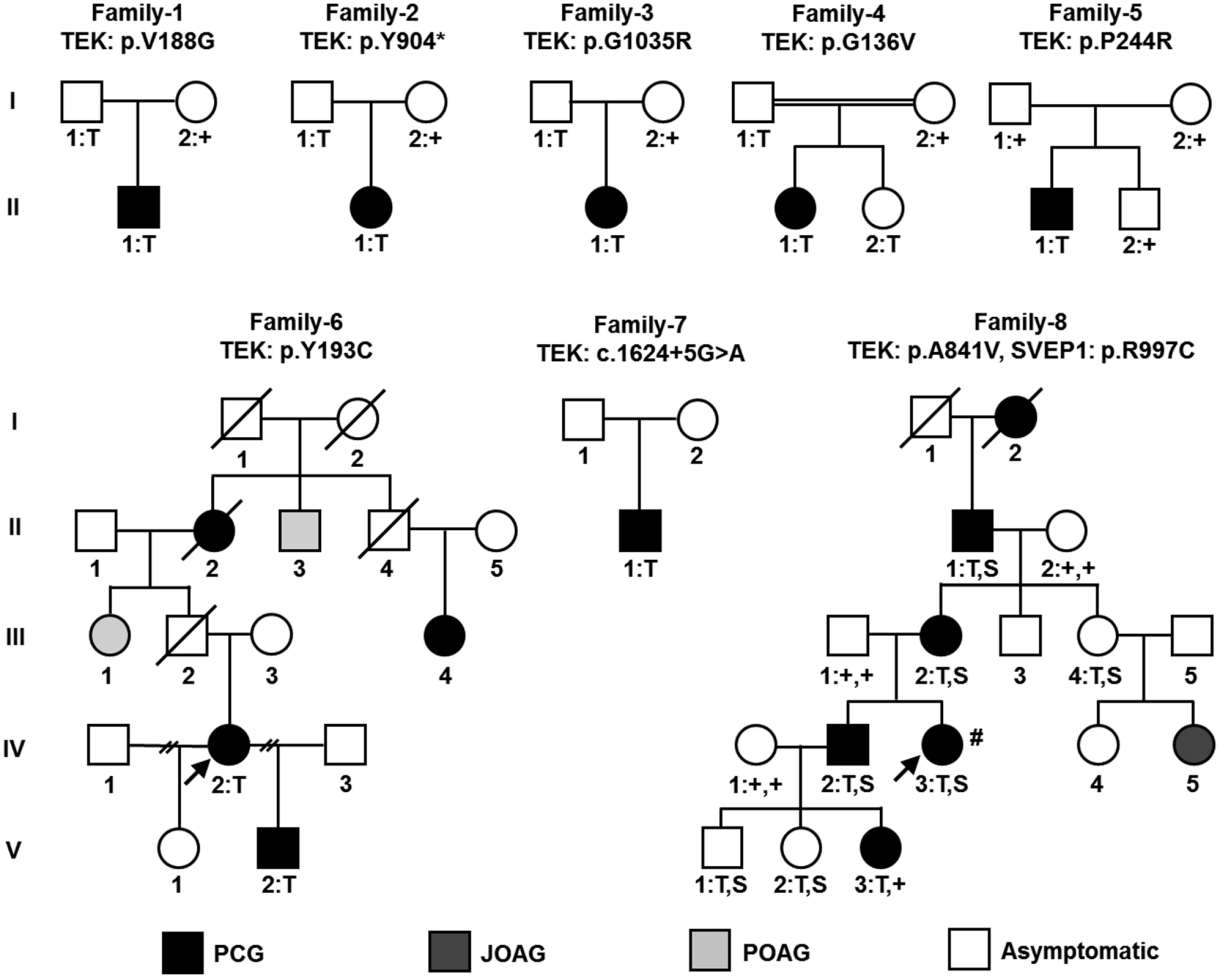
Pedigrees of PCG families one through eight. Phenotypic presentations are shown for PCG (*black shaded*), juvenile open-angle glaucoma (JOAG, *dark grey shaded*), primary open-angle glaucoma (POAG), (*light grey shaded*), and asymptomatic (*unshaded*). For participating family members, the genotypes are given below each individual: heterozygous *TEK* variant, T; heterozygous *SVEP1* variant, S; reference genotype, +. Probands in multi-affected families are denoted by arrows. An ocular pathology sample was obtained from the individual denoted by #.

**Figure 2.**
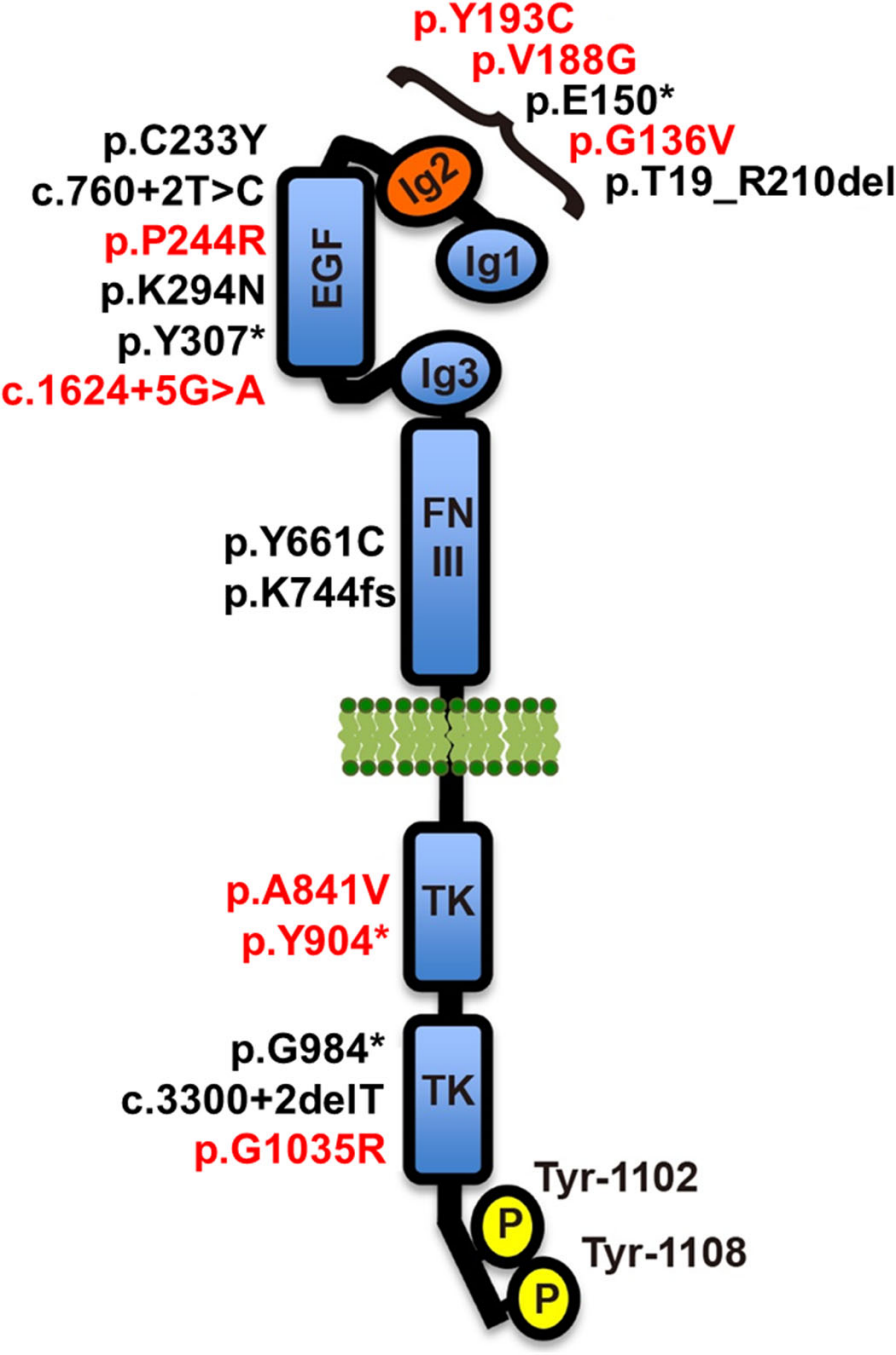
A diagrammatic representation of the TEK receptor displaying the locations of 18 variants we have identified to date. Eight variants identified in this study are highlighted in red, and 10 variants shown in black were previously reported.^13^ The receptor contains three immunoglobulin-like domains (Ig1, Ig2, and Ig3) and an epidermal growth factor-like domain (EGF) that are involved in ligand binding, a fibronectin type-III domain (FNIII), a single-pass transmembrane domain (cell membrane lipid bilayer represented in *green*), and an interrupted protein kinase domain (TK) within the intracellular C-terminal tail. The primary tyrosine phosphorylation sites, Tyr-1102 and Tyr-1108, are shown at the C-terminal tail, which are responsible for initiating downstream signaling events. *TEK* mRNA reference sequence: NM_000459.4. TEK protein reference sequence: NP_000450.2.

Exome sequencing identified eight PCG families with rare, heterozygous variants in the *TEK* gene. The pedigrees for these families are provided (Fig. 1), with ocular clinical details (Table 1), gene variant information (Table 2), and the protein location of each variant (Fig. 2). Nonocular clinical features were noted in several members of family 8, including ovarian cysts, Legg-Calvé-Perthes disease (osteochondrosis of the hip joint), and gastroparesis (Supplementary Table S2).

**Table 1.** Ocular Clinical Information for PCG Families 1 Through 8

Individual	Glaucoma	Onset age	Cup–Disk Ratio	Surgery/Treatments	Other
F1-II1	PCG (OD)	<1 yo	1.0 (PS)	3 angle surgeries	Megalocornea (diameter 14 mm, 10 yo), IOP 35 mm Hg (10 yo), 30 mm Hg (PS)
F2-II1	PCG (OS)	3 months	0.1/0.3 (PS)	Goniotomy (OS), trabeculectomies (2 OS)	Haab striae, IOP 17 mm Hg (PS)
F3-II1	PCG (OU)	<1 yo	0.3/0.3 (PS)	1 surgery (OU)	IOP 21/22 mm Hg (PS)
F4-II1	PCG (OU)	4 months	0.2/0.2 (PS)	Goniotomy, trabeculectomies (2 OS)	Enlarged corneas (13.5/13 mm), corneal edema with clouding, Haab striae, IOP 19/22.5 mm Hg (PS), parents 1^st^ cousins once removed
F5-II1	PCG (OD)	4 months	0.8 (OD)	Topical beta-blocker medication (timolol)	Buphthalmos, IOP 15 mm Hg (with timolol)
F6-II2	PCG	Childhood			
F6-II3	POAG	70s			
F6-III1	POAG	50s			
F6-III4	PCG	4 yo			
F6-IV2	PCG (OU)	2 yo	0.8 (OD)	Enucleation (OS), goniotomy (OU), trabeculotomies (2 OS, 1 OD)	Scleral staphyloma (OS)
F6-V2	PCG (OU)	4 months	0.6/0.5	Tube shunt, diode cyclophotocoagulation (OD)	Iris posterior synechiae (OD), scleral staphyloma (OD)
F7-II1	PCG (OU)	Birth			
F8-I2	PCG (OU)	<5 yo		Enucleation (OU)	
F8-II1	PCG (OU)			Enucleation (OD)	Posterior vitreous detachment
F8-III2	PCG (OU)	8 months	0.3 (OS)	Trabeculectomies (OU), superior sector iridectomy (OS), tube shunt (OD), enucleation (OD, 24 yo)	OS, 38 yo: IOP 23 mm Hg, posterior vitreous detachment, bleb epithelial overgrowth (trabeculectomy), cataract, visual acuity light perception only
F8-IV2	PCG (OS)	4 yo		Multiple goniotomies/trabeculectomies (OS)	
F8-IV3	PCG (OU)	3 months		Multiple goniotomies/cyclocryotherapies (OS), trabeculectomy (OS), double plate Molteno implant (OS), Molteno implant (OD), enucleation (OD), revision of Molteno implant (OS)	IOP 30/30 mm Hg (10 yo), retinal detachment (OD), ocular pathology sample; moderate corneal band keratopathy (OS), cataract with posterior iris synechiae (OS), Enlarged cornea (diameter 13 mm, OS), visual acuity 20/400 (OS), IOP 10 mm Hg (OS, 28 yo) following ciliary body destruction
F8-IV5	JOAG				Astigmatism, strabismus, cornea problems
F8-V3	PCG (OU)	18 months		Goniotomy, multiple surgeries	

PCG, primary congenital glaucoma; POAG, primary open-angle glaucoma; JOAG, juvenile open-angle glaucoma; OU, both eyes; OS, left eye; OD, right eye; yo, years old; PS, post-surgery.

**Table 2.** *TEK* and *SVEP1* Variants Identified in Eight PCG Families

Family ID	Ethnicity	Gene	Chromosome Position	Exon	Coding DNA Variant	Protein Alteration	Variant Count: Total Alleles^a^	Variant Count: ETHNICALLY Matched Alleles^b^
1	European (Portugal)	*TEK*	9:27169562	4	c.563T>G	p.V188G	0 : 251,260	0 : 113,570
2	European (Australia)	*TEK*	9:27212730	17	c.2712C>G	p.Y904*	0 : 251,242	0 : 113,590
3	European (Australia)	*TEK*	9:27218815	20	c.3103G>C	p.G1035R	0 : 251,376	0 : 113,694
4	South Asian (Iran)	*TEK*	9:27168535	3	c.407G>T	p.G136V	0 : 251,176	0 : 30,610
5	South Asian (Iran)	*TEK*	9:27172716	5	c.731C>G	p.P244R	0 : 251,198	0 : 30,608
6	European (America)	*TEK*	9:27169577	4	c.578A>G	p.Y193C	0 : 251256	0 : 30,616
7	European (America)	*TEK*	9:27192626	IVS11	c.1624+5G>A	p.Val497_Ile541del	1 : 250,382	0 : 112,886
8	European (America)	*TEK*	9:27206737	15	c.2522C>T	p.A841V	1 : 251,142	1 : 113,500
		*SVEP1*	9:113233653	16	c.2989C>T	p.R997C	4 : 248,368	2 : 112,418

Chromosome position in accordance with GRCh37/hg19 assembly. *TEK* mRNA reference sequence NM_000459.4. TEK protein reference sequence NP_000450.2. *SVEP1* mRNA reference sequence NM_153366.4. SVEP1 protein reference sequence NP_699197.3. Variant allele frequency data acquired from the Genome Aggregation Database (gnomAD, 2.1.1 release, 6 Mar 2019).

^a^Number of times the variant allele has been observed versus the total number of alleles sequenced in the global population at that genome position.

^b^Number of times the variant allele has been observed versus the total number of alleles sequenced in an ethnically matched population at that genome position.

All eight *TEK* variants were rare in the gnomAD database, a compilation of exome/genome variants from over 140,000 individuals. Six *TEK* variants were novel, and two were each seen in only one individual (Table 2). Because selection keeps deleterious gene variants at a low population frequency, these rare *TEK* variants were considered to be detrimental to the gene's function. The p.Y904* nonsense variant was located in exon 17 of the 23 exon gene, and expected to result in degradation of the transcript via nonsense-mediated decay. The consequence of the splice site variant (c.1624+5G>A) was assessed *in silico* using Human Splicing Finder (HSF), which predicted the donor splice site to be nonfunctional. All six missense changes involved residues that have been evolutionarily conserved for 360, 400, or 450 million years, since humans shared a common ancestor with the western clawed frog, coelacanth, or spotted gar, respectively (see Supplementary Fig. S1). All affected residues were located within important protein regions as determined by crystallography (see Supplementary Fig. S2), and all were predicted by *in silico* tools to impact protein function and stability (Table 3). In addition, the p.G1035R substitution resulted from a nucleotide change at the last position of exon 20 (c.3103G>C, NM_000459.4), a location important for donor splice site recognition. Analysis using HSF, predicted the base change to result in a nonfunctional splice site.

**Table 3.** Functional Predictions of Residue Substitution's Pathogenicity, Sequence Conservation Scores, and Free Energy (Protein Stability) Changes

Method	TEK p.G136V	TEK p.V188G	TEK p.Y193C	TEK p.P244R	TEK p.A841V	TEK p.G1035R	SVEP1 p.R997C
SIFT	Damaging	Damaging	Damaging	Damaging	Damaging	Damaging	Damaging
PolyPhen-2	Probably damaging	Probably damaging	Probably damaging	Probably damaging	Probably damaging	Probably damaging	Probably damaging
MutationTaster	Disease causing	Damaging	Disease causing	Disease causing	Damaging	Disease causing	Damaging
LRT	Deleterious	Deleterious	Deleterious	Deleterious	Deleterious	Unknown	Deleterious
MutationAssessor	Predicted nonfunctional (low)	Tolerated	Predicted functional (medium)	Predicted functional (medium)	Predicted functional (medium)	Predicted functional (medium)	Predicted functional (medium)
FATHMM	Tolerated	Tolerated	Tolerated	Tolerated	Damaging	Tolerated	Tolerated
MetaSVM	Damaging	Damaging	Damaging	Tolerated	Damaging	Damaging	Tolerated
MetaLR	Damaging	Damaging	Damaging	Tolerated	Damaging	Damaging	Tolerated
CADD^a^	26.6	26.4	27.6	26.8	29.8	34	32.0
PhyloP^b^	6.3	5.5	4.0	6.1	5.4	2.4	4.6
GERP++^c^	5.6	5.5	5.5	5.1	5.9	4.8	5.4
FoldX^d^	4.45 kcal/mol (SD: 0.05)	4.27 kcal/mol (SD: 0.03)	2.13 kcal/mol (SD: 0.05)	4.96 kcal/mol (SD: 2.11)	1.83 kcal/mol (SD: 0.03)	14.43 kcal/mol (SD: 1.00)	N/A

a CADD PHRED-scaled scores are normalized to the potential 9 billion single nucleotide variations (SNVs) in human genome; a score of 10 or greater indicates an estimated pathogenicity in the top 10% of all SNVs, 20 or greater indicates within the top 1%, and 30 or greater within the top 0.1%.

b PhyloP basewise conservation score derived from Multiz alignment of 100 vertebrate species; positive scores show conservation, which is slower evolutionary drift than expected.

c GERP++ rejected substitutions is the number of substitutions expected under genomic evolutionary neutrality minus the number of substitutions “observed” at the position; scores range from 1.00 to 6.18; higher scores demonstrate a more conserved site.

d FoldX average ddG (difference in free energies between mutant and WT protein) run over 5 times. PDB structure 4K0V (p.G136V, p.V188G, p.Y193C, p.P244R) and 1FVR (p.A841V, p.G1035R) used as templates; N/A, PDB crystal structure unavailable. TEK protein reference sequence: NP_000450.2. SVEP1 protein reference sequence: NP_699197.3.

In all eight PCG families, no rare variants except for those observed in *TEK* were identified in genes previously associated with PCG or later onset forms of glaucoma.

### *TEK* Variants Represent LoF Alleles

**Figure 3.**
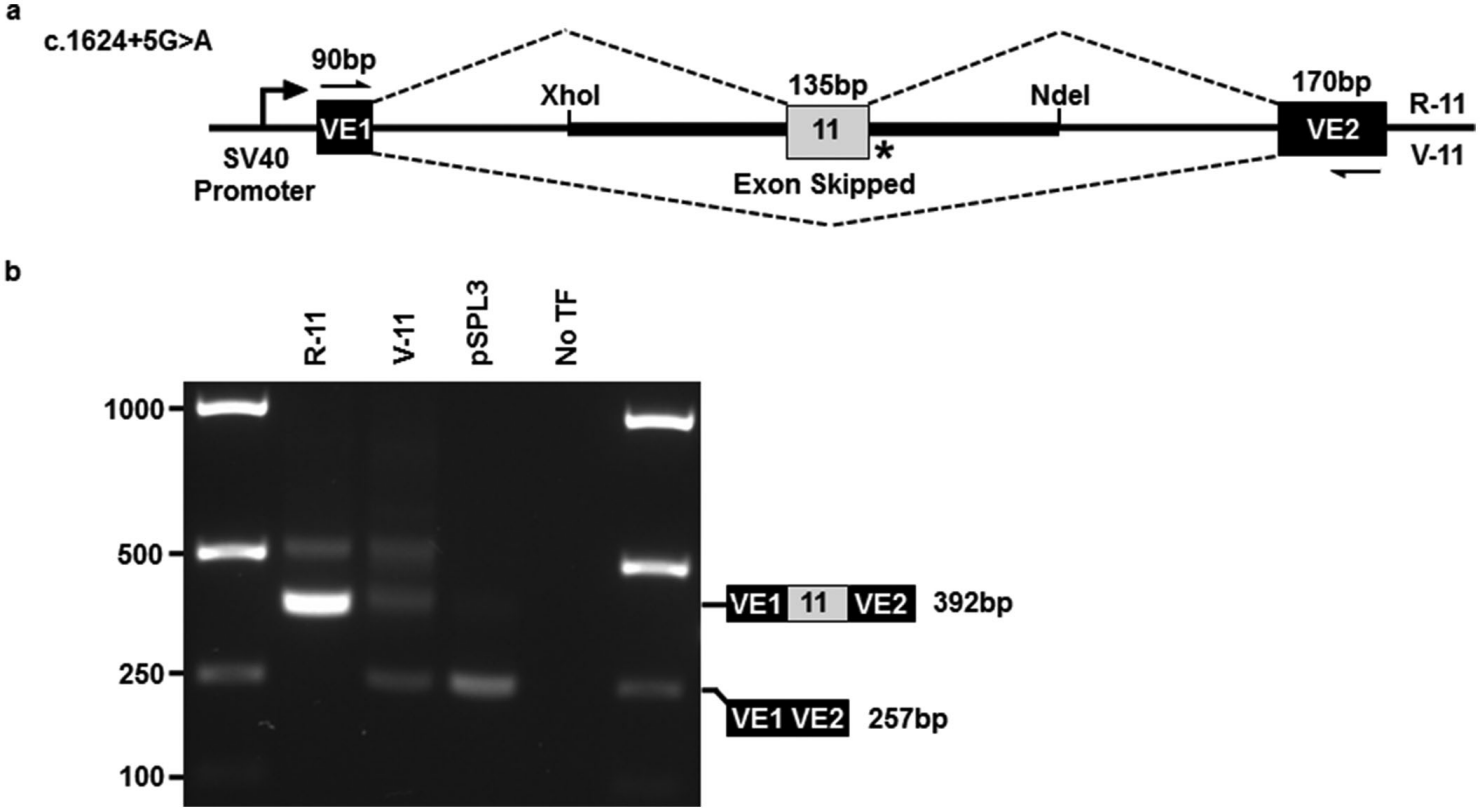
Exon trapping of the *TEK* c.1624+5G>A donor splice site variant leads to skipping of exon 11. (**a**) Reference and variant mini-genes were generated by incorporating genomic regions of *TEK* gene exon 11 into the pSPL3 exon trapping vector via XhoI and NdeI restriction sites. Vector exons VE1 and VE2 are depicted as black boxes and *TEK* exon 11 is in gray. Reference (R) and variant (V) splicing products are indicated by dashed lines above and below the construct, respectively. The location of the splice site variant is shown (*). (**b**) Gel electrophoresis of RT-PCR products from transfected Cos-7 cells, using vector exon-specific primers (indicated by half-arrows in a). pSPL3, cDNA template from cells transfected with empty pSPL3 vector only; No TF, cDNA template from cells transfected without plasmid DNA. *TEK* mRNA reference sequence: NM_000459.4.

In a cell-based exon trapping assay, the c.1624+5G>A splice site variant destroyed the donor splice site, leading to skipping of exon 11 (Fig. 3). *TEK* transcripts lacking exon 11 encode a receptor with an in-frame deletion of 45 amino acids (Val497-Ile541, NP_000450.2) which form parts of the immunoglobulin-like fold (Ig3) and fibronectin type III (FNIII) domains. As these domains are integral for ligand binding, we determine the splice variant allele to be LoF.

**Figure 4.**
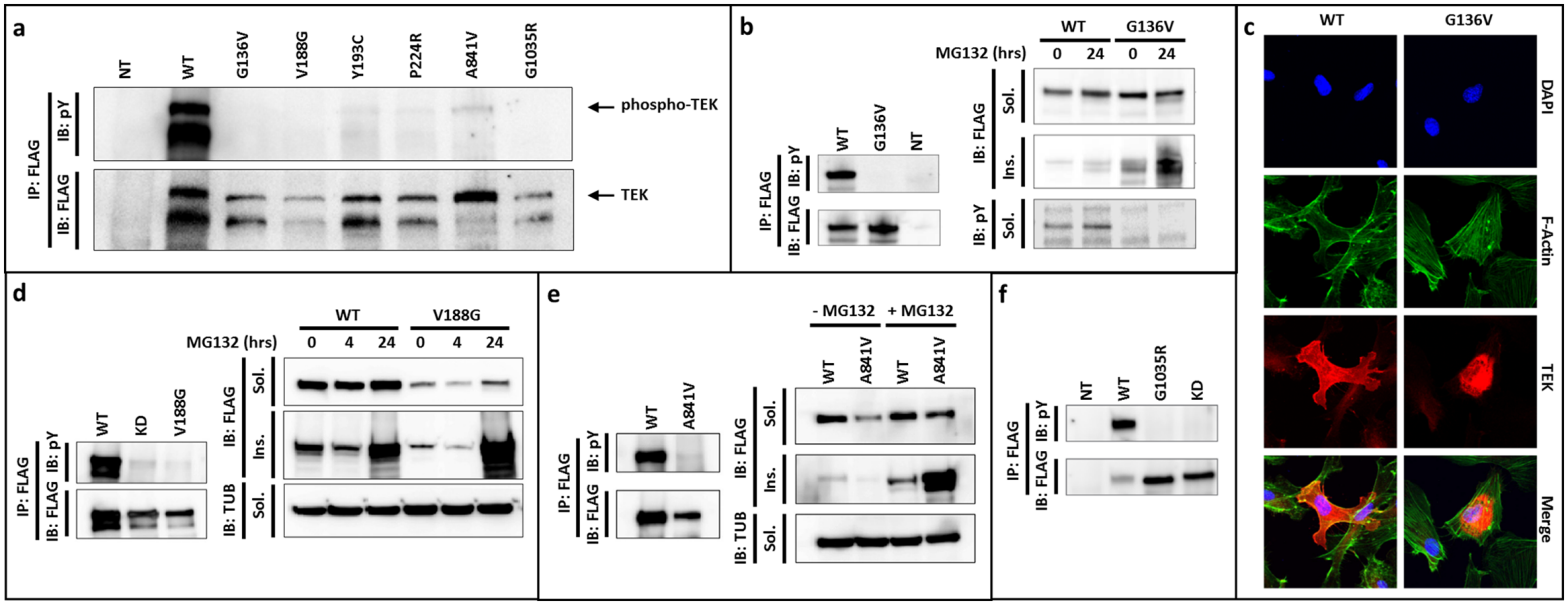
Functional assessment of TEK missense protein expression, receptor activation, solubility, proteasomal degradation, and cell localization. (**a**) WT and mutant TEK protein levels were detected by FLAG-tag immunoprecipitation and blotting (lower blot), while the extent of receptor phosphorylation was detected with an antibody against phospho-tyrosine (pY, upper blot). Strong phospho-TEK levels were detected with WT receptor, but all six mutant proteins failed to phosphorylate. (**b**) *Left*, G136V receptor levels were comparable to WT, but phosphorylation was undetectable. *Right*, G136V protein showed increased insolubility that was more apparent following proteasome inhibition. (**c**) Immunofluorescence microscopy detected WT receptors at the cell membrane and throughout the cytoplasm, but G136V protein remained within a likely proteasome-associated cytoplasmic region. (**d**) *Left*, V188G receptors failed to autophosphorylate, similar to the KD mutant. *Right*, V188G protein was detected at low levels, but identified as a strong insoluble component when proteasome degradation was inhibited. (**e**) A841V receptors showed undetectable phosphorylation (*left*), and accumulation in the insoluble component after proteasome inhibition (*right*). (**f**) G1035R receptors also failed to phosphorylate. IP, immunoprecipitation; IB, immunoblot; FLAG, anti-FLAG; pY, anti-phosphotyrosine; TUB, anti-α-tubulin loading control; MG132, proteasome inhibitor; NT, not transfected; Sol., soluble fraction; Ins., insoluble fraction; KD, TEK protein lacking kinase domain. TEK protein reference sequence: NP_000450.2.

TEK receptors containing each of the six missense substitutions were overexpressed in HEK293T cells and their solubility levels, cellular localization, proteasomal degradation, and functional activity were compared with WT receptors (Fig. 4). TEK normally localizes to the plasma membrane, and its tyrosine kinase activity can be detected via autophosphorylation of its C-terminal tyrosine residues. All six missense variant receptors were unable to autophosphorylate, similar to the KD mutant harboring a nonfunctional kinase domain (Fig. 4). The six variant proteins also showed lower expression intensities, suggesting instability or degradation of the abnormal receptors. After 24 hours of MG132 proteasome inhibition, p.G136V-, p.V188G-, and p.A841V-variant proteins were strongly detected in the insoluble fraction, unlike their WT counterpart. In addition, immunofluorescence microscopy showed that although WT-TEK localized throughout the cytoplasm and plasma membrane, the p.G136V mutant remained localized to a perinuclear cytoplasmic region, likely associated with proteasomes. These findings demonstrated that all six missense variants resulted in nonfunctional TEK receptors, and indicated the mutant proteins are likely degraded via the proteasome pathway.

Altogether, the functional studies revealed that, along with the nonsense change (p.Y904*), all eight *TEK* variant alleles were LoF, further supporting receptor haploinsufficiency as a cause of PCG.

### Ocular Pathology From Family 8 Revealed Absence of Aqueous Outflow Structures

**Figure 5.**
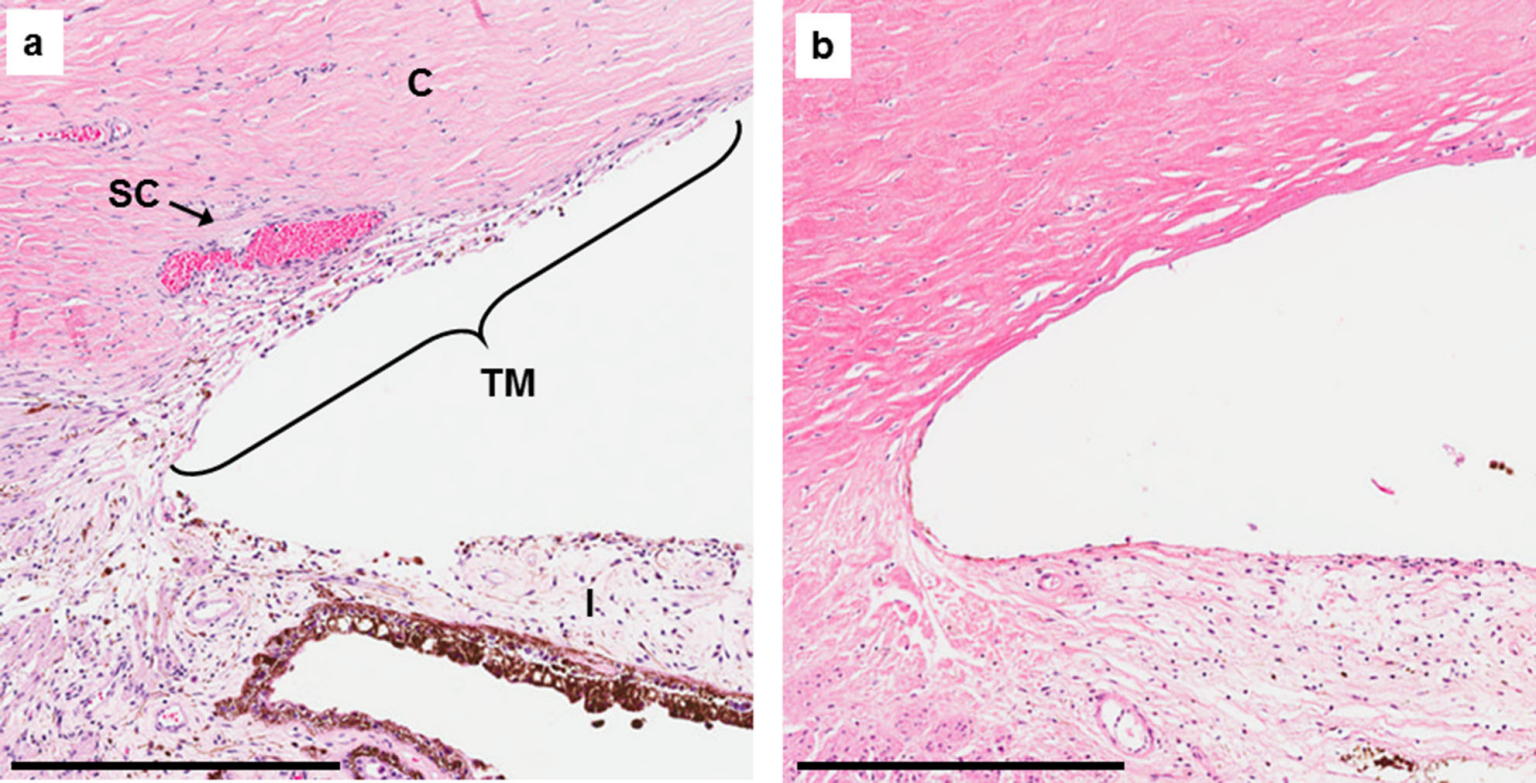
Ocular pathology of iridocorneal angles from an age-matched control without ocular disease (**a**) and a PCG-affected individual from family 8 (**b**). Histologic sections stained with hematoxylin and eosin. Note: SC is blood-filled in the control eye owing to physical trauma. C, cornea; I, iris; TM, trabecular meshwork; SC, Schlemm 10s canal. Scale bar = 400 µm.

Histologic examination of the ocular outflow pathway was possible for an affected member of family 8 (Fig. 1, family 8, individual IV-3, #), an 11-year-old with PCG refractory to multiple surgeries. Comparison with an unrelated, disease-free, 11-year-old eye was also possible owing to an enucleation after a BB gun accident (Fig. 5). In the eye lacking disease, and hematoxylin and eosin staining clearly revealed the TM and SC, which was blood-filled owing to the injury. In the affected eye, the anterior chamber showed a blunted iridocorneal angle, with no evidence of TM or SC. Additionally, the sclera was mildly hypercellular, and the scleral spur was not evident. Descemet's membrane did not end abruptly, but showed an ambiguous tapering further into the angle to a region where the TM should be located. To highlight SC, tissue sections were immunostained for the EC marker CD31, but no evidence of SC was observed in the PCG-affected angle (data not shown).

### Additional *SVEP1* Variant Identified in Family 8

Exome sequencing 3 affected individuals (III-2, IV-2 and IV-3) from family 8 identified additional rare variants in the *SVEP1* gene (p.R997C, rs761025824; Table 2) and nine other genes (Supplementary Table S3). Sanger sequencing confirmed the SVEP1 heterozygous missense change in four of the five participating affected individuals, but it was not carried by the youngest affected female (Fig. 1). The other nine gene variants did not cosegregate with the glaucoma phenotype. In gnomAD, the SVEP1-p.R997C variant was observed in four alleles globally, and *in silico* analyses predicted the substitution to be damaging (Table 3). Comparative protein sequence alignments revealed conservation of Arginine-997 over 450 million years, since humans and spotted gar shared a common ancestor (see Supplementary Fig. S1).

### SVEP1 Is Expressed by Periocular Mesenchyme Surrounding the Developing SC

**Figure 6.**
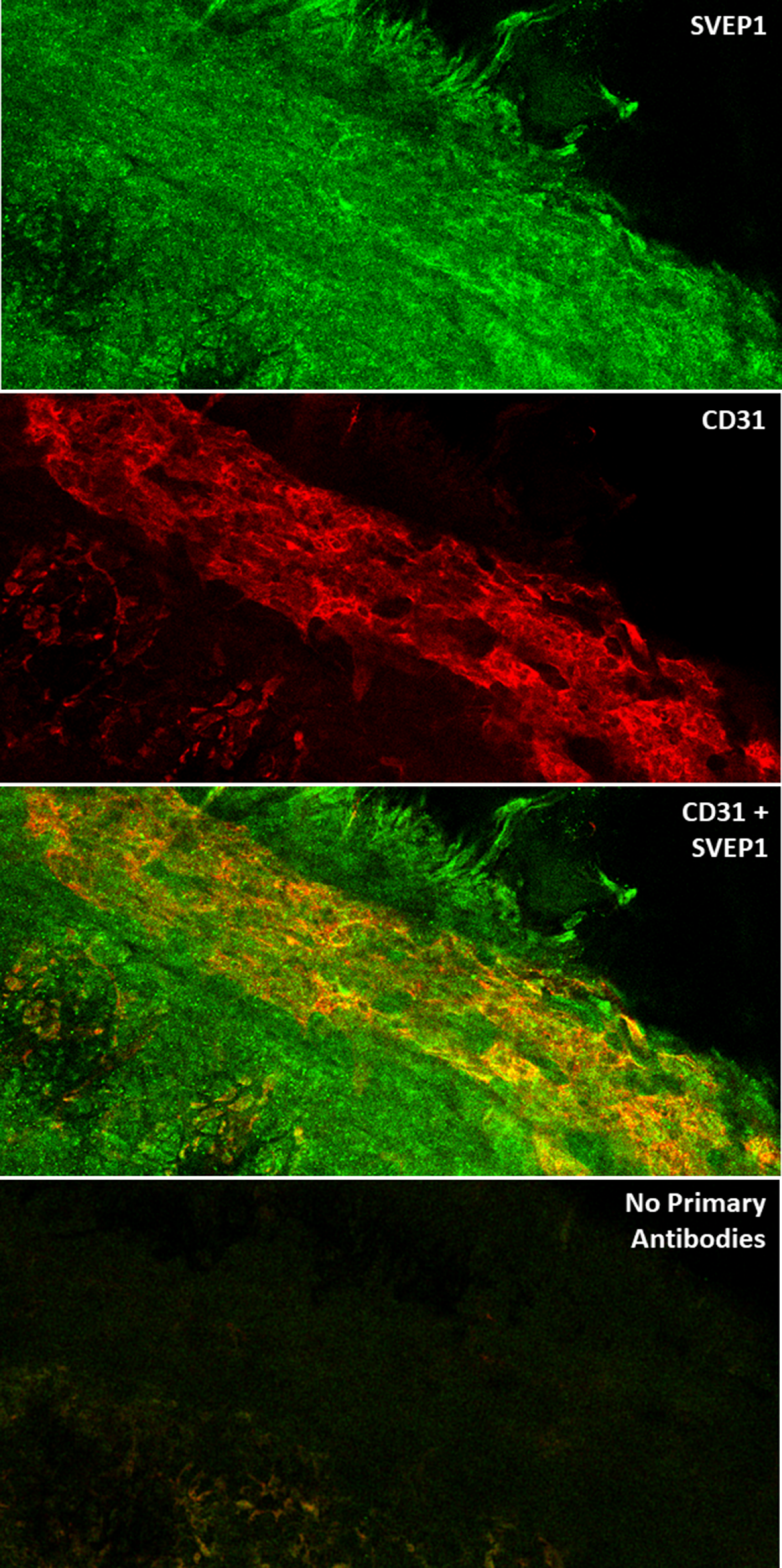
Immunofluorescent confocal imaging of flat-mounted 1-week old mouse anterior segments stained for SVEP1 and CD31 protein. (**a**) SVEP1 (*green*) expression is observed in the mesenchyme of the developing iridocorneal angle. (**b**) CD31 (*red*), an EC marker, is detected within the interwoven branches of the maturing SC and the ancillary vasculature. (**c**) Overlay revealed mesenchymal-expressed SVEP1 protein in close proximity to the developing SC. (**d**) Anterior segments processed without the addition of SVEP1 and CD31 primary antibodies showed minimal background signal from the secondary antibodies alone.

Immunofluorescent confocal imaging of SVEP1 and CD31 expression in flat-mounted 1-week-old mouse anterior segments identified the developing SC as an interwoven clustering of branched vessels adjacent to the limbus (Fig. 6). Strong SVEP1 staining was detected throughout the periocular mesenchyme and in close proximity to the ECs of the maturing SC.

### *SVEP1* Missense Variant Impairs Exogenous Stimulation of Endothelial *TEK* Expression

**Figure 7.**
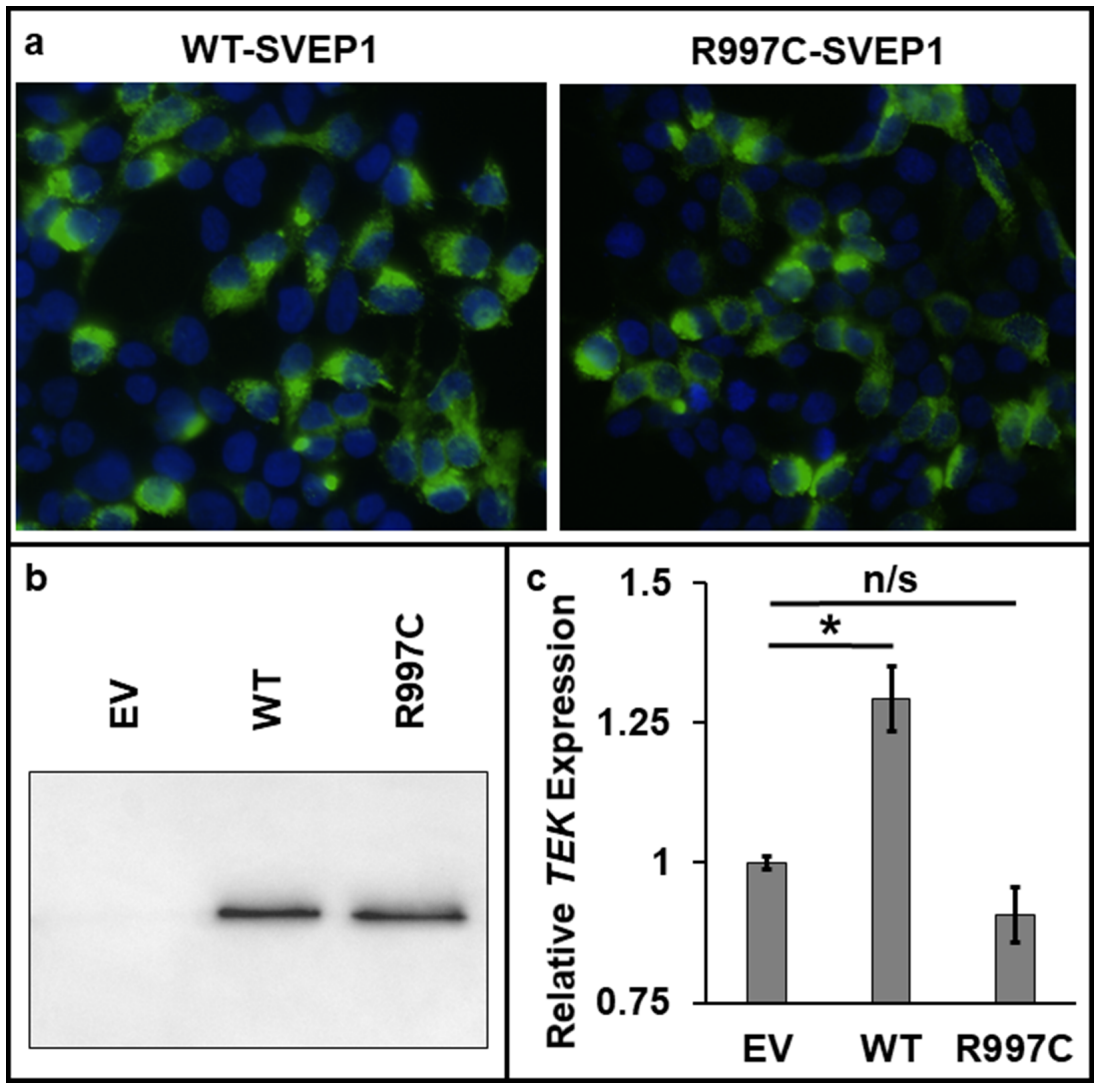
Functional assessment of WT and p.R997C-SVEP1. (**a**) Immunostaining (*green*) of WT- or p.R997C-SVEP1 expressed in transfected HEK293T cells. Nuclei are counterstained with DAPI (*blue*). (**b**) Immunoblot of conditioned medium from HEK293T cells expressing WT-SVEP1, p.R997C-SVEP1, or an empty FLAG vector (EV). (**c**) TaqMan quantitative real-time PCR analysis showing fold change of *TEK* gene expression in HUVECs treated with WT-SVEP1- or p.R997C-SVEP1-conditioned medium relative to untreated (empty FLAG vector) cells. Error bars show the mean standard error over 10 samples per condition. Statistically significant differences between pairs of conditions are denoted (*P < 0.05; n/s, not significant). SVEP1 protein reference sequence: NP_699197.3.

Immunofluorescence microscopy of cultured HEK293T cells expressing WT- and p.R997C-variant SVEP1 constructs revealed comparable levels of protein localized to the cytoplasm (Fig. 7A). Western blotting identified equivalent levels of WT and variant SVEP1 in the conditioned media, indicating both proteins were similarly secreted (Fig. 7B). To evaluate the stimulatory effect of exogenous SVEP1 on EC expression of *TEK*, HUVECs were treated with SVEP1-conditioned medium and *TEK* expression measured by TaqMan assay. HUVECs treated with WT-SVEP1 showed a moderate but consistent upregulation of *TEK* expression compared with untreated cells (1.29 fold change, *P* = 0.001; Fig. 7C and Dataset 1). In contrast, p.R997C-SVEP1 treatment consistently failed to enhance *TEK* expression (0.91 fold change, *P* = 0.139), which revealed the p.R997C variant abrogated the exogenous signaling function of SVEP1.

## Discussion

We report eight PCG families with rare heterozygous LoF variants in the *TEK* gene, supporting a causative role for *TEK* in PCG. More extensive pedigrees could be ascertained for two families, which together contained ten individuals affected by PCG. Parent-to-child disease transmission was observed in both families, providing strong evidence in support of an autosomal dominant mode of inheritance. *TEK* variants were also identified in several asymptomatic individuals, an observation that is consistent with reduced disease penetrance, and may be explained by the development of variably hypomorphic SCs in carrier individuals, as has been shown for hemizygous mice.^13^ Reduced penetrance has also been reported for PCG caused by LoF variants in *CYP1B1*.^10^^,^^24^^–^^26^ Notably, in a study of 22 Saudi kindreds, 40 asymptomatic individuals had *CYP1B1* variants identical to their affected siblings.^24^ An analysis of the pedigrees suggested the presence of a dominant modifier locus that suppressed the disease phenotype.

Compared with the 17 other families identified with functionally-confirmed *TEK*-related PCG, family 8 was remarkable in several aspects. Their disease penetrance was strikingly higher, with affected individuals observed in five consecutive generations. Furthermore, 83% (5/6) of the affected individuals presented with disease in both eyes, whereas *TEK*-related PCG has been bilateral in 50% (10/20) of the affected individuals identified to date. Additionally, the disease in this family seemed to be more refractory to treatment, with four individuals requiring enucleations despite multiple attempts at surgical intervention to control IOP. Histologic examination of one individual's enucleated eye (Fig. 1, family 8, individual IV-3, #) revealed an apparent absence of SC and TM, comparable to murine eyes lacking a functional *Tek* gene.^13^ Interestingly, a number of nonocular clinical features were also noted in several family members both with and without glaucoma, including four with ovarian cysts, three with Legg-Calvé-Perthes disease, and two suffering from gastroparesis. All five participating individuals affected with PCG were heterozygous for a *TEK* LoF variant, of which four had an additional rare variant in the *SVEP1* gene that was anticipated to be functionally deleterious. However, because the *SVEP1* variant was not carried by the youngest affected individual (V-3), it was not necessary for PCG manifestation. Nonetheless, given the extraordinary features of this family, we hypothesized that the *SVEP1* variant could play a role as a disease modifier.

In zebrafish and mice, SVEP1 was found to be essential for lymphatic vessel formation.^27^^,^^28^ Zebrafish embryos that lacked *svep1* had aberrant venous sprouting and migration of lymphatic ECs (LECs).^27^ Similarly in mice, loss of *Svep1* caused abnormal lymphovenous connections and lymphatic remodeling, which resulted in dysfunctional fluid drainage and severe embryonic edema.^27^ During normal development, SVEP1 is secreted from mesenchymal cells and deposited around adjacent LECs, where it activates lymphatic remodeling via forkhead box c2 (FOXC2) signaling.^28^ Of relevance, dermal LECs isolated from mice lacking SVEP1 showed reduced expression of *Foxc2* and *Tek*, implicating these genes as downstream effectors of SVEP1 signaling.^28^ Furthermore, other studies have shown that FOXC2 can promote *TEK* expression in mouse and human LECs.^21^ Together, these studies reveal that, during lymphatic vessel development, SVEP1 acts as an extracellular signaling messenger to stimulate FOXC2 expression in ECs, which in turn upregulates expression of TEK.

In humans, we note that within the University of Washington's shared web resource, Geno2MP (https://geno2mp.gs.washington.edu/Geno2MP), exome sequencing has identified five additional *SVEP1* missense variants in patients with glaucoma (variant details provided in Supplementary Table S4). All five alleles were rare and had CADD scores over 25, further supporting an association between *SVEP1* gene variants and glaucoma.

In consideration of these findings, we hypothesized that the additional *SVEP1*:p.R997C variant in family 8 may act to further decrease TEK receptor levels during SC development, a specialized hybrid vessel that displays molecular markers of both lymphatic and venous ECs. In mouse, SC begins to form at birth when ECs sprout and migrate out from blood vessels at the limbus, and by 1 week a rudimentary vessel exists around the eye.^6^ During this period, the adjacent tissue of the future iridocorneal angle is composed of a densely packed periocular mesenchyme.^29^^,^^30^ Immunofluorescent imaging of 1-week-old mouse anterior segments detected strong SVEP1 staining in the periocular mesenchymal tissues surrounding the interwoven vessels of the developing SC, consistent with SVEP1 performing a similar role in SC formation as has been shown for lymphatic development.

When HUVECs were grown in medium conditioned with WT-SVEP1, a moderate but consistent upregulation of *TEK* expression was stimulated, demonstrating exogenous SVEP1 can enhance vascular EC expression of *TEK* in this *in vitro* system. In contrast, medium conditioned with p.R997C-SVEP1 failed to promote *TEK* expression, confirming the missense variant negated this aspect of the protein's function.

Together, the results suggest that the observed increase in disease penetrance and severity in family 8 likely resulted from limited TEK signaling within the developing SC. Consequently, these individuals would be expected to show increased SC dysgenesis, which is consistent with the observed ocular pathology. It should be noted that several members of family 8 shared additional nonocular clinical features, such as Perthes disease and ovarian cysts, which may also be related to abnormal SVEP1 and/or TEK signaling.

PCG is a devastating ocular disorder that affects infants within the first 3 years of life. Although the initial ocular hypertension is known to be caused by inadequate aqueous humor outflow, the underlying molecular and cellular disease mechanisms remain poorly understood. Herein, we provide molecular genetic findings that support *TEK* haploinsufficiency as a cause of PCG, and affirm the autosomal dominant inheritance pattern in two extended multi-affected pedigrees. For the first time, we report evidence underscoring a role for *SVEP1* as a modifier of *TEK* expression in vascular ECs, and propose SVEP1 as a modifier of *TEK*-related PCG disease penetrance and severity. These findings provide a potential new molecular target for directed therapeutic drug intervention in congenital glaucoma.

## Supplementary Material

Supplement 1

Supplement 2
