# Supplementary material for: SVEP1 as a Genetic Modifier of TEK-Related Primary Congenital Glaucoma
Source: Invest Ophthalmol Vis Sci. 2020 Oct 7;61(12):6. doi: 10.1167/iovs.61.12.6 (PMC7545080; doi:10.1167/iovs.61.12.6)
Supplement: Supplement 2 [file iovs-61-12-6_s002.pdf]

SUPPLEMENTARY MATERIAL

SVEP1 as a Genetic Modifier of *TEK*-Related Primary Congenital Glaucoma

Terri L. Young*, Kristina N. Whisenhunt*, Jing Jin*, Sarah M. LaMartina, Sean M. Martin, Tomokazu Souma, Vachiranee Limviphuvadh, Fatemeh Suri, Emmanuelle Souzeau, Xue Zhang, Yongwook Dan, Evie Anagnos, Susana Carmona, Nicole M. Jody, Nickie Stangel , Emily C. Higuchi, Samuel J. Huang, Owen M. Siggs, Maria José Simões, Brendan M. Lawson, Jacob S. Martin, Elahe Elahi, Mehrnaz Narooie-Nejad, Behzad Fallahi Motlagh, Susan E. Quaggin, Heather D. Potter, Eduardo D. Silva, Jamie E. Craig, Conceição Egas, Reza Maroofian, Sebastian Maurer-Stroh, Yasmin S. Bradfield, Stuart W. Tompson#

1 Department of Ophthalmology and Visual Sciences, University of Wisconsin-Madison, Madison, Wisconsin, USA.

2 Feinberg Cardiovascular Research Institute and Division of Nephrology/Hypertension, Northwestern University Feinberg School of Medicine, Chicago, Illinois, USA.

3 Bioinformatics Institute (BII), Agency for Science Technology and Research (A*STAR), Singapore.

4 Innovations in Food & Chemical Safety Programme (IFCS), A*STAR, Singapore.

5 Ophthalmic Research Center, Shahid Beheshti University of Medical Sciences, Tehran, Iran.

6 Department of Ophthalmology, Flinders University, Flinders Medical Centre, Adelaide, South Australia, Australia.

7 Biocant, Transfer Technology Association, Cantanhede, Portugal.

8 Faculty of Medicine, University of Coimbra, Coimbra, Portugal.

9 School of Biology, University College of Science, University of Tehran, Tehran, Iran.

10 Genetics of Non-communicable Disease Research Center, Zahedan University of Medical Science, Zahedan, Iran.

11 Nikukari Eye Hospital, Tabriz University of Medical Sciences, Tabriz, Iran.

12 Faculty of Medicine, Institute for Biomedical Imaging and Life Sciences, University of Coimbra, Coimbra, Portugal.

13 Center for Neuroscience and Cell Biology, University of Coimbra, Coimbra, Portugal.

14 Genetics Research Center, Molecular and Clinical Sciences Institute, St George's, University of London, Cranmer Terrace, London, UK.

15 Department of Biological Sciences, National University of Singapore (NUS), Singapore.

* Terri L. Young, Kristina N. Whisenhunt, and Jing Jin should be considered joint first authors.

# Please address all correspondence to: Stuart W. Tompson, PhD; Department of Ophthalmology and Visual Sciences, University of Wisconsin-Madison, WIMR2 9551, 1111 Highland Ave, Madison, WI 53705, USA; E-mail: stompson@wisc.edu; Phone: 1-608-265-8052.

SUPPLEMENTARY MATERIALS AND METHODS

Exome Sequencing and Variant Filtering

Venous blood, saliva or buccal mucosal samples were collected from eight unrelated families affected by PCG (Figure 1). Genomic DNA samples were exome sequenced at independent institutions using several capture kits and sequencing platforms. The proband from Family-1 was sequenced using an Ion AmpliSeq Exome Kit (Thermo Fisher Scientific, Waltham, MA) and an Ion Proton System (Thermo Fisher Scientific). Variants were annotated with wANNOVAR.[1] Exome sequencing of probands from Family-2 and Family-3 was achieved by way of a Sure Select Human All Exon Kit (version 5; Agilent), 100bp paired-end reads, and a HiSeq 4000 platform (Illumina). All four individuals from Family-4 and the proband from Family-5 were exome sequenced via a commercial service (Novogene, Chula Vista, CA), which utilized a Sure Select Human All Exon Kit (version 6; Agilent, Santa Clara, CA), 150bp paired-end reads, and a HiSeq 2500 platform (Illumina, San Diego, CA). The proband from Family-6 was whole-genome sequenced via a commercial service (Hudson Alpha, Huntsville, AL) using paired-end reads and a HiSeq X platform (Illumina). The proband from Family-7 was exome sequenced in 2011 via Hudson Alpha, utilizing a SeqCap EZ Exome Library v2.0 kit (Roche NimbleGene, Madison, WI), 90bp paired-end reads, and a HiSeq 2000 platform (Illumina). Three affected and three unaffected individuals from Family-8 were exome sequenced at the University of Wisconsin Biotechnology Center DNA Sequencing Facility (Madison, Wisconsin) utilizing a Sure Select Human All Exon Kit (version 5; Agilent), 100bp paired-end reads, and a HiSeq 2000 platform (Illumina).

Sequencing reads were mapped to the human reference genome assembly (NCBI build 37/hg19), and exome variants analyzed using SNP and Variation Suite (SVS) software version 8.4 (Golden

Helix, Bozeman, Montana). Sequence changes located within exonic and splice site regions (-3 bp to +6 bp) were selected for further analysis. Variants with a global allele frequency greater than 0.0001 in the Genome Aggregation Database (gnomAD v2.1.1; https://gnomad.broadinstitute.org)[2] were excluded from further analysis. Missense variants were evaluated according to evolutionary conservation of the affected residue using GERP++ (Genomic Evolutionary Rate Profiling)[3] and PhyloP (100-way vertebrate).[4] *In silico* predictions of pathogenicity were generated using the programs SIFT[5], Polyphen-2[6], MutationTaster[7], Likelihood Ratio Test (LRT)[8], MutationAssessor[9], Functional Analysis Through Hidden Markov Models (FATHMM)[10], radial kernel Support Vector Machine (MetaSVM)[11], Logistic Regression (LR)[12], and Combined Annotation-Dependent Depletion (CADD).[13] The effect of residue substitution on protein stability was assessed using FoldX (http://foldx.embl.de).[14] For templates, the Protein Data Bank (PDB, http://www.rcsb.org) crystal structures for the human TEK ectodomain region complexed with Angiopoietin-1 (PDB ID: 4K0V) and the human TEK kinase domain region (PDB ID: 1FVR) were utilized. The functional effect of splice site variants were predicted using Human Splicing Finder (HSF v3.1; http://www.umd.be/HSF/HSF.shtml).[15]

In all eight PCG families, no rare variants except for those observed in *TEK* were identified in genes associated with PCG or later-onset forms of glaucoma. Aside from the *TEK* and *SVEP1* gene variants, exome sequence analysis of the three affected individuals (III-2, IV-2 and IV-3) from Family-8 identified nine additional low allele frequency gene variants (Supplementary Table S3). None of these genes have been previously associated with PCG, and none of the variants co-segregated with the glaucoma phenotype.

Variant Confirmation by Sanger Sequencing

Gene variants were confirmed in each subject by direct Sanger sequencing using primers listed in Supplementary Table S1. Sequencing electropherograms were aligned to the Ensembl GRCh37 genomic reference sequences for *TEK* (ENSG00000120156) and *SVEP1* (ENSG00000165124) using Vector NTI Suite 8.0 (Thermo Fisher) or Sequencher 5.2.4 (Gene Codes Corporation, Ann Arbor, MI) software. Gene variants were annotated with their corresponding protein changes according to *TEK* transcript NM_000459.4 (NP_000450.2) and *SVEP1* transcript NM_153366.4 (NP_699197.3).

Confocal Immunofluorescence Microscopy of Mouse Anterior Segment Flat Mounts

Eyes from 1-week old wild-type 129S1/SvImJ mice were enucleated and immersion fixed with cold 2% PFA in 1x phosphate buffered saline (PBS), pH 7.4, at 4°C for 24 hours. The eyes were washed in cold PBS containing 150 mM glycine and 0.1% Tween 20 for 1 hour, followed by 3x 5 minute washes with cold PBS. Eyes were stored in 70% ethanol (molecular biology grade) at 4°C until processed further. Eyes were dissected on a PBS-soaked Kimwipe to avoid drying and to aid manipulation. The conjunctiva and extra-ocular tissues were removed, and an incision was made through the sclera, just posterior to the limbus, to separate the anterior eye cup. The retina, vitreous and lens were removed, and the remaining eye cup placed into a 5 ml conical polypropylene tube. The tissue was blocked for 16 hours at 4°C with 5% bovine serum albumin solution (BSA), 5% normal goat serum, and 1x Tris-buffered saline (pH 7.5) containing 0.5% Triton X100 (TBS-T) and 0.01% NaN_3 (blocking buffer, BB). The tissue was incubated with rat anti-mouse CD31 antibody (1:50; Cat # 553370, BD Pharmingen) and rabbit anti-human SVEP1 antibody (1:250; Cat # PA5-54436, Thermo Fisher) in fresh BB for 48 hours at 4°C with gentle rocking. Control tissues were also processed without the addition of primary antibodies. The eye

cups were washed 3x 1 hour in TBS containing 0.05% Tween 20 (wash buffer, WB) at room temperature (RT), and then incubated with a goat anti-rat IgG Alexa Fluor 594 secondary antibody (1:1000; Cat # A-11007, Thermo Fisher) and a goat anti-rabbit IgG Alexa Fluor 488 secondary antibody (1:300; Cat # ab150081, Abcam) in BB for 48 hours at 4°C. The eye cups were washed 6x 1 hour in WB at RT and then transferred to a glass coverslip with the cornea facing down. Using fine spring-scissors, a series of centripetal cuts were made towards the center of the cornea, creating a flattened flower petal shape. The tissue was coverslip mounted with Shandon Immu-Mount (Thermo Fisher) and covered overnight at 4°C. Next, the slides were returned to RT to allow condensate to evaporate and then sealed with clear fingernail polish. Imaging was performed on a Nikon A1RS confocal microscope. For low-magnification images of Schlemm's canal, the confocal pinhole was set to 150μm, and a series of stitched images was captured using a 20x objective. Depth projections were prepared from confocal Z-stacks captured using a 20x objective and a 20 μm pinhole (1.2 Aery units). The imaging plane was focused on the superficial perilimbal vasculature and 44 μm Z-stacks towards the center of the eye were captured.

The specificity of the anti-SVEP1 antibody was also verified via immunohistochemical staining of human placenta sections (Supplementary Figure S3).

Functional Assessment of *SVEP1* Missense Variant

A WT-SVEP1 expression construct was generated by cloning full-length (10.7 kb) *Svep1* cDNA into the pSF-CMV-NEO-COOH-3XFLAG vector (Sigma-Aldrich Corp., St. Louis, MO) in-frame with the 3'-FLAG tag. Placental tissue from a pregnant 129S1/SvImJ inbred mouse was used as a source of full-length Svep1 mRNA (highest expression in The Human Protein Atlas; https://www.proteinatlas.org/). A construct expressing *SVEP1*-p.R997C was generated by site-

directed mutagenesis. Constructs were transfected into subconfluent 293FT cells (Thermo Fisher Scientific) using FuGENE 6 (Promega, Madison, WI), and grown in DMEM with high glucose, 6mM L-glutamine, 10% FBS and 1% P/S at 37ºC and 5% CO_2. SVEP1 expression was analyzed by Western blot. Briefly, after 24 hours, cells were lysed using Laemmli Sample Buffer (LSB) with 2.5% 2-ME, and passed through a 21-guage needle to shear genomic DNA. Proteins were denatured at 95°C for 5 minutes, loaded into 4-20% TGX gels (Bio-Rad), separated by denaturing electrophoresis, and transferred to PVDF membranes. Anti-FLAG M2 antibody (1:1000) was used to detect FLAG-tagged SVEP1 proteins and anti-GAPDH (1:1000; Cell Signaling Technology, Danvers, MA) as a loading control. To assess SVEP1 secretion, cells were grown in serum-free media for 2 days, the conditioned media collected, cell components removed by centrifugation, and Western blotting performed.

SVEP1 localization was studied in HEK293T cells grown to subconfluence on glass cover slips and transfected with Svep1 expression constructs. After 24 hours, cells were fixed with 4% PFA for 15 minutes at RT, blocked and permeabilized with 5% BSA with 0.3% Triton X100 in PBS for 1 hour at RT, and incubated overnight at 4°C with anti-FLAG-FITC antibody (1:500; M2, F4049, Sigma-Aldrich). Cells were washed 3 x 5 minute with PBS, and mounted with ProLong Diamond antifade mountant with DAPI (Thermo Fisher Scientific). Images were captured using an Olympus BX51 fluorescence microscope.

To evaluate the effect of exogenous SVEP1 on *TEK* gene expression in HUVECs (PCS-100-013; ATCC, Manassas, VA), constructs expressing WT-SVEP1, p.R997C-SVEP1, and empty FLAG vector (EV) were expressed in HEK293T cells. After 24 hours, cells were washed with PBS and the medium replaced with VCBM (PCS-100-030; ATCC) supplemented with an Endothelial Cell Growth Kit-VEGF (PCS-100-041; ATCC). After 2 days, the conditioned media was collected

and cell components removed by centrifugation. Aliquots of conditioned media were frozen in LSB with 2.5% 2-ME for subsequent Western blot analysis, and the remaining conditioned media added to growing HUVECs. After 24 hours, cells were lysed and processed using the Cells-to-cDNA II kit (Thermo Fisher). *TEK* gene expression was assessed by quantitative real-time PCR using a pre-developed TaqMan assay probe (ID: Hs00176096_m1, FAM-MGB; Thermo Fisher) and TaqMan Fast Advanced Master Mix (Thermo Fisher) in accordance with the manufacturer's instructions on an ABI Quant Studio 7 Flex RT-PCR cycler (Applied Biosystems). The expression of beta-actin (human *ACTB* endogenous control, FAM/MGB probe, non-primer limited; Thermo Fisher) was used as an internal RNA normalization control. Real-time PCR reaction conditions were as follows: pre-denaturation at 95°C for 20 seconds, followed by 40 cycles of denaturation at 95°C for 1 second, and annealing at 60°C for 20 seconds. Three independent experiments were performed totaling 10 biological samples for each condition, and 4 technical replicates were averaged for each sample. *TEK* expression fold change in cells treated with WT- or p.R997C-SVEP1 was calculated relative to untreated (EV) cells using the $2^{-\Delta\Delta Ct}$ method.[16] Statistical significance between the means of condition pairs were assessed using the two-tailed t-test. The standard deviation of each test condition was corrected to account for the three experiment means used to pool the data.

Supplementary References

1. Yang H, Wang K. Genomic variant annotation and prioritization with ANNOVAR and wANNOVAR. *Nat Protoc* 2015;10:1556-1566.
2. Karczewski KJ, Francioli LC, Tiao G, et al. Variation across 141,456 human exomes and genomes reveals the spectrum of loss-of-function intolerance across human protein-coding genes. *bioRxiv* 2019.
3. Davydov EV, Goode DL, Sirota M, Cooper GM, Sidow A, Batzoglou S. Identifying a high fraction of the human genome to be under selective constraint using GERP++. *PLoS Comput Biol* 2010;6:e1001025.
4. Pollard KS, Hubisz MJ, Rosenbloom KR, Siepel A. Detection of nonneutral substitution rates on mammalian phylogenies. *Genome Res* 2010;20:110-121.

5. Kumar P, Henikoff S, Ng PC. Predicting the effects of coding non-synonymous variants on protein function using the SIFT algorithm. *Nat Protoc* 2009;4:1073-1081.
6. Adzhubei IA, Schmidt S, Peshkin L, et al. A method and server for predicting damaging missense mutations. *Nat Methods* 2010;7:248-249.
7. Schwarz JM, Rodelsperger C, Schuelke M, Seelow D. MutationTaster evaluates disease-causing potential of sequence alterations. *Nat Methods* 2010;7:575-576.
8. Chun S, Fay JC. Identification of deleterious mutations within three human genomes. *Genome Res* 2009;19:1553-1561.
9. Reva B, Antipin Y, Sander C. Predicting the functional impact of protein mutations: application to cancer genomics. *Nucleic Acids Res* 2011;39:e118.
10. Shihab HA, Gough J, Cooper DN, et al. Predicting the functional, molecular, and phenotypic consequences of amino acid substitutions using hidden Markov models. *Hum Mutat* 2013;34:57-65.
11. Kim S, Jhong JH, Lee J, Koo JY. Meta-analytic support vector machine for integrating multiple omics data. *BioData Min* 2017;10:2.
12. Liu X, Wu C, Li C, Boerwinkle E. dbNSFP v3.0: A One-Stop Database of Functional Predictions and Annotations for Human Nonsynonymous and Splice-Site SNVs. *Hum Mutat* 2016;37:235-241.
13. Kircher M, Witten DM, Jain P, O'Roak BJ, Cooper GM, Shendure J. A general framework for estimating the relative pathogenicity of human genetic variants. *Nat Genet* 2014;46:310-315.
14. Schymkowitz J, Borg J, Stricher F, Nys R, Rousseau F, Serrano L. The FoldX web server: an online force field. *Nucleic Acids Res* 2005;33:W382-388.
15. Desmet FO, Hamroun D, Lalande M, Collod-Beroud G, Claustres M, Beroud C. Human Splicing Finder: an online bioinformatics tool to predict splicing signals. *Nucleic Acids Res* 2009;37:e67.
16. Livak KJ, Schmittgen TD. Analysis of relative gene expression data using real-time quantitative PCR and the 2(-Delta Delta C(T)) Method. *Methods* 2001;25:402-408.

Supplementary Table S1: Primer pairs for PCR amplification and Sanger sequencing of *TEK* and *SVEP1* gene variants.

Variant	Forwards Primer (5' to 3')	Reverse Primer (5' to 3')
TEK p.G136V[a]	AGTGCCAGCCCTCATTTTC	GCCCACAAGACCACAATAGG
TEK p.V188G[a]	CTTGACCATGTCAGGGAAAGC	ATACAACCTCCAACCAATGCCT
TEK p.Y193C[a]	CTTGACCATGTCAGGGAAAGC	ATACAACCTCCAACCAATGCCT
TEK p.P244R[a]	TTCACCATTGTCCACTGAATG	TAGATCCAGCAACGATGGC
TEK c.1624+5G>A[b]	ATCGCAATAACAACAACCCC	CCTGGTCTTCCTTCCTCTTTC
TEK p.A841V[a]	ACCAGAAGACATTATGCCCC	AAATCAAGTTTTCTCCACACCC
TEK p.Y904*[a]	CACAGCACATCTCTTAAATG	GAGAGGTTGACAGACCCTAC
TEK p.G1035R[a]	TGTAGACATTTAACATCTCC	CATCTGGTAGAACCACATAC
SVEP1 p.R997C[c]	ACAACCACACAACAAGAGCA	AGCATCTGAGAGGACTGAAACA

a, TEK protein reference sequence NP_000450.2; b, TEK transcript reference sequence NM_000459.4; c, SVEP1 protein reference sequence NP_699197.3.

Supplementary Table S2: Additional ocular and extra-ocular clinical information for individual members of Family-8.

Individual	Ocular	Musculo-skeletal	Reproductive	Cardiovascular/ Hematopoietic	Gastro-intestinal	Other
F8-I2	PCG (OU), enucleation (OU)	Perthes	Multiple miscarriages			Esophageal cancer
F8-II1	PCG (OU), enucleation (OD), posterior vitreous detachment (OS), eyelid cyst			Heart issues		
F8-II2	Cataracts					Hypertension, hypercholesterolemia
F8-III2	PCG (OU), enucleation (OD), posterior vitreous detachment (OS)	Perthes, reconstruction of right lower leg, chronic ankle pain, C5 perineural cysts	Ovarian cysts, endometriosis (25 yo), several miscarriages	Heart murmur, chronic venous insufficiency		Herniated disk, partial hysterectomy, hearing loss (antibiotics), cystic kidneys
F8-III3	Cataracts	Perthes, gout				
F8-III4			Ovarian cysts			Breast cancer, diabetes, pituitary gland tumor
F8-IV2	PCG (OS)					Hearing loss (chronic infections)
F8-IV3	PCG (OU), extreme photophobia, cataract (OU), enucleation (OD), band keratopathy, macular edema, retinal detachment, iris synechiae (OS)	Arthritis, scoliosis, Ehlers-Danlos syndrome-hypermobility type, fibromyalgia	Ovarian cysts	Heart murmur, heart attack at 3 months, Blood clots	Gastroparesis	Lyme disease, kidney stones, seizures, migraines
F8-IV4			Ovarian cysts	Blood clots	Gastroparesis	
F8-IV5	JOAG, astigmatism, strabismus, cornea problems					
F8-V1						Speech impediment, born 28 weeks

PCG, primary congenital glaucoma; JOAG, juvenile open-angle glaucoma; OU, both eyes; OS, left eye; OD, right eye; yo, years old.

Supplementary Table S3: Additional low allele frequency gene variants identified in three affected individuals (III-2, IV-2 and IV-3) from Family-8.

Gene Name	Genomic Location	Protein Change
VIT	2:37035700 G>A	p.Arg492Gln
CYB561D2	3:50391057 C>T	p.Thr184Ile
COL14A1	8:121301955 G>A	p.Val1396Met
ZNF250	8:146108049 C>G	p.Gln178His
KIAA1161	9:34372341 C>A	p.Asp167Tyr
TTC40	10:134740157 A>C	p.Leu327Arg
EIF4G2	11:10822589 C>T	p.Val487Met
KIAA0556	16:27781340 G>A	c.4133+1G>A (splice)
EWSR1	22:29682919 T>A	p.Ser203Thr

Supplementary Table S4: *SVEP1* missense variants identified in the Geno2MP database in 5 individuals with a "glaucoma" phenotype.

Genomic Location	dbSNP ID	Coding DNA Variant	Protein Alteration	gnomAD Global Allele Frequency	Conserve Conservation Score	GERP Score	PolyPhen-2 Prediction	CADD Score
9:113137660 G>A	rs368027292	c.10588C>T	p.(R3530C)	0.0001	1	5.45	Probably Damaging	34
9:113168807 C>T	rs73530980	c.9073G>A	p.(G3025R)	0.0004	1	5.76	Probably Damaging	31
9:113173587 G>C	rs201520602	c.6404C>G	p.(S2135C)	0.0019	1	5.74	Probably Damaging	26.2
9:113233652 C>T	rs61732547	c.2990G>A	p.(R997H)	0.0006	0.879	5.49	Probably Damaging	35
9:113265470 G>A	rs116347456	c.1331C>T	p.(P444L)	0.0017	1	5.71	Benign	26.1

Chromosome position in accordance with GRCh37/hg19 assembly. SVEP1 mRNA reference sequence NM_153366.4. SVEP1 protein reference sequence NP_699197.3. Variant allele frequency data acquired from the Genome Aggregation Database (gnomAD, 2.1.1 release, 6 Mar 2019).

Links to Geno2MP glaucoma-associated variants in *SVEP1*:

https://geno2mp.gs.washington.edu/Geno2MP/#/variant/9/113137660/G%253EA/snp
https://geno2mp.gs.washington.edu/Geno2MP/#/variant/9/113168807/C%253ET/snp
https://geno2mp.gs.washington.edu/Geno2MP/#/variant/9/113173587/G%253EC/snp
https://geno2mp.gs.washington.edu/Geno2MP/#/variant/9/113233652/C%253ET/snp
https://geno2mp.gs.washington.edu/Geno2MP/#/variant/9/113265470/G%253EA/snp

	T-G136	T-V188	T-Y193	T-P244	T-A841	T-G1035	S-R997
Human	VDKGDNV	DAGVYSA	SARYIGG	ICPPGFM	VLKARIK	VSLGGTP	LRGRMCV
Chimp	VDKGDNV	DAGVYSA	SARYIGG	ICPPGFM	VLKARIK	---GGTP	LRGRMCV
Gorilla	VDKGDNV	DAGVYSA	SARYIGG	ICPPGFM	VLKARIK	VSLGGTP	LRGRMCV
Orangutan	VDKGDNV	DAGVYSA	SARYIGG	ICPPGFM	-------	VSLGGTP	LRGRMCV
Gibbon	VDKGDNV	DAGVYSA	SARYIGG	ICPPGFM	VLKARIK	VSLGGTP	LRGRMCV
Rhesus	VDKGDNV	DAGVYSA	SARYIGG	ICPPGFM	VLKARIK	VSLGGTP	-------
Baboon	VDKGDNV	DAGVYSA	SARYIGG	ICPPGFM	VLKARIK	VSLGGTP	LRGRMCV
Marmoset	VDKGDNV	DAGVYSA	SARYIGG	ICPPGFM	VLKARIK	VSLGGTP	LRGRMCV
Chinese tree shrew	VDRGDNV	DAGVYSA	SARYIGG	ICPPGFM	VLKARIK	VSLGGTP	LRGRMCV
Squirrel	VDRGDNV	DAGVYSA	SARYIGG	ICPPGFM	VLKARIK	VSLGGTP	LRGRMCV
Chinese hamster	VDRGDNV	DAGVYSA	SARYIGG	ICPPGFM	VLKARIK	VSLGGTP	LRGRMCV
Mouse	VDRGDNV	DAGVYSA	SARYIGG	ICPPGFM	VLKARIK	VSLGGTP	LRGRMCV
Rat	VDRGDNV	DAGVYSA	SARYIGG	ICPPGFM	VLKARIK	VSLGGTP	LRGRMCV
Guinea pig	VNKGDNV	DAGVYSA	SARYIGG	ICPPGFM	VLKARIK	VSLGGTP	LRGRMCV
Rabbit	VDKGDTV	DAGVYSA	SARYIGG	ICPPGFM	VLKARIK	VSLGGTP	LRGRMCV
Pig	VDRGDNV	DAGVYSA	SARYIGG	ICPPGFM	VLKARIK	VSLGGTP	LRGRMCV
Cow	VDRGDNV	DAGVYSA	SARYIGG	ICPPGFM	VLKARIK	VSLGGTP	LRGRMCV
Sheep	VDRGDNV	DAGVYSA	SARYIGG	ICPPGFM	VLKARIK	VSLGGTP	LRGRMCV
Domestic goat	VDRGDNV	DAGVYSA	SARYIGG	ICPPGFM	VLKARIK	VSLGGTP	LRGRMCV
Horse	VDRGDNV	DAGVYSA	SARYIGG	ICPPGFM	VLKARIK	VSLGGTP	LRGRMCV
Cat	VDRGDNV	DAGVYSA	SARYIGG	ICPPGFM	VLKARIK	VSLGGTP	LRGRMCV
Dog	VDRGDNV	DAGVYSA	SARYIGG	ICPPGFM	VLKARIK	VSLGGTP	LRGRMCV
Elephant	VDKGDNV	DAGVYSA	SARYIGG	ICPPGFM	VLKARIK	VSLGGTP	LRGRMCV
Aardvark	VDRGDNV	DAGVYSA	SARYIGG	ICPPGFM	VLKARIK	VSLGGTP	LRGRMCV
Armadillo	VDRGDNV	DAGVYSA	SARYIGG	ICPPGFM	VLKARIK	VSLGGTP	LRGRMCV
Platypus	VNKGENV	DSGVYSA	SARYIGG	-------	VLKARIK	VSLGGTP	LRGRMCV
Chicken	ANKGEHV	DAGVYSA	SARYIGG	ICPPGFM	VLKARIK	VSLGGTP	LKGRMCV
American alligator	ANKGESV	DAGVYYA	YARYIGG	ICPPGFM	VLKARIK	VSLGGTP	LRGRMCV
Green sea turtle	VNKGEHV	DAGVYSA	SARYIGG	ICPPGFM	VLKARIK	VSLGGTP	LRGRMCV
Lizard	ANKRDDV	DAAVYSA	SARYIGG	ICPPGFM	VLKARIK	VSLGGTP	LRGRMCV
Western clawed frog	ASKGDNT	DASVYRV	RVTYMGG	ICPPGFM	VLKARIK	VSLGGTP	LKGRMCV
Coelacanth	VNKGENV	DAGVYTA	TARFIGG	ICPPGFM	VLKARIK	VSLGGTP	LKGRMCV
Tetraodon	-------	-------	-------	-------	V------	VSLGGTP	LKGRMCV
Fugu	-------	-------	-------	-------	V---R-K	---GGTP	LNGRMCV
Stickleback	-------	-------	-------	ICPPGFM	V-K--IK	VSLGGTP	LKGRMCV
Atlantic cod	-------	-------	-------	-------	--K--IK	VSLGGTP	-------
Zebrafish	-------	-------	-------	-------	V-K--IK	VSLGGTP	LKGRVCV
Mexican tetra	-------	-------	-------	VCPPGFM	V-K--IK	---GGTP	LKGRMCV
Spotted gar	-------	DSGFYSV	SVNFIAG	ICPPGFM	V-KARIK	VSLGGTP	LKGRMCV
Lamprey	-------	-------	-------	-------	-------	-------	-------

Supplementary Figure S1: Conservation of orthologous protein sequences at TEK (T) and SVEP1 (S) missense locations. Multiple sequence alignments across 40 vertebrate species are given for 7 regions containing TEK Gly-136, Val-188, Tyr-193, Pro-244, Ala-841, Gly-1035, and SVEP1 Arg-997. Conserved residues are highlighted at the missense location in red and adjacent locations in blue. Non-conserved residues are not highlighted, and missing sequence data is noted by dashed lines. Data obtained from UCSC Genome Browser, Vertebrate Multiz Alignment & Conservation (100 Species), human genome assembly GRCh37/hg19 (Feb, 2009). TEK protein reference sequence: NP_000450.2. SVEP1 protein reference sequence NP_699197.3.

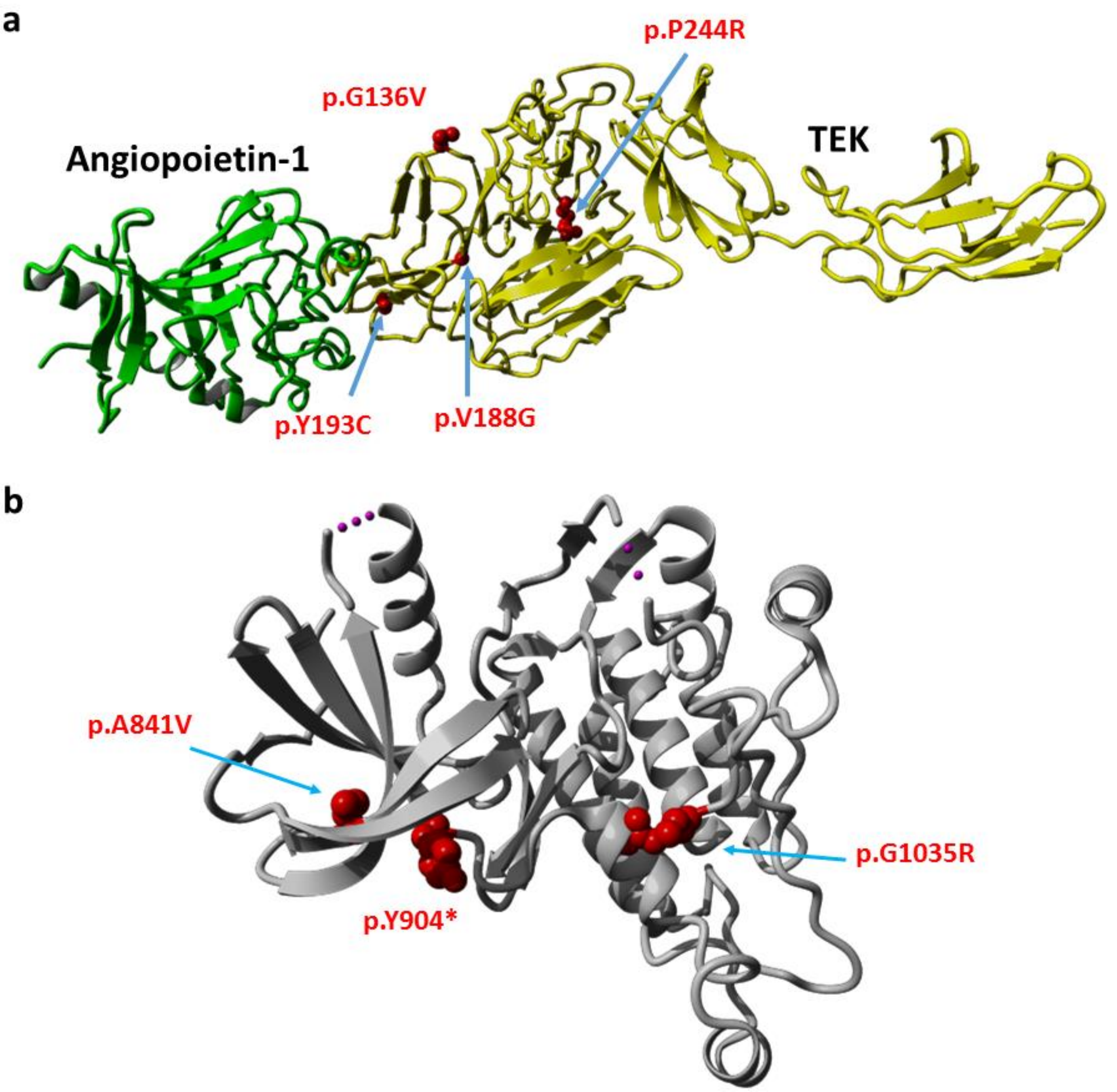

Supplementary Figure S2: Crystal structures of human TEK protein regions highlighting the locations of the 6 missense variants. a) Crystal structure of the human TEK ectodomain region (yellow, PDB: 4K0V, 4.51 Å resolution) complexed with the primary ligand Angiopoietin-1 (green), showing missense variants p.G136V, p.V188G, p.Y193C, and p.P244R (red). p.G136V, p.V188G and p.Y193C are located within the ligand-binding Ig2 domain. p.G136V substitutes a surface exposed glycine to a hydrophobic valine, and may indirectly affect the ligand binding site. p.V188G is within a beta-sheet of the Ig2 domain, and substitution of the hydrophobic valine may affect the core stability. Substitution of proline-244 (nonpolar) to an arginine (basic) in the core is likely to affect conformation. b) Crystal structure of the human TEK kinase domain region (grey, PDB: 1FVR, 2.2 Å resolution), showing missense variants p.A841V and p.G1035R (red). The location of the p.Y904* variant is also shown. p.A841V is located within a beta-sheet of the kinase domain, whereas p.G1035R lies within a core flexible loop region. The substitution of glycine-1035 to a positively charged arginine is likely to interfere with the stability of the core region. TEK protein reference sequence: NP_000450.2.

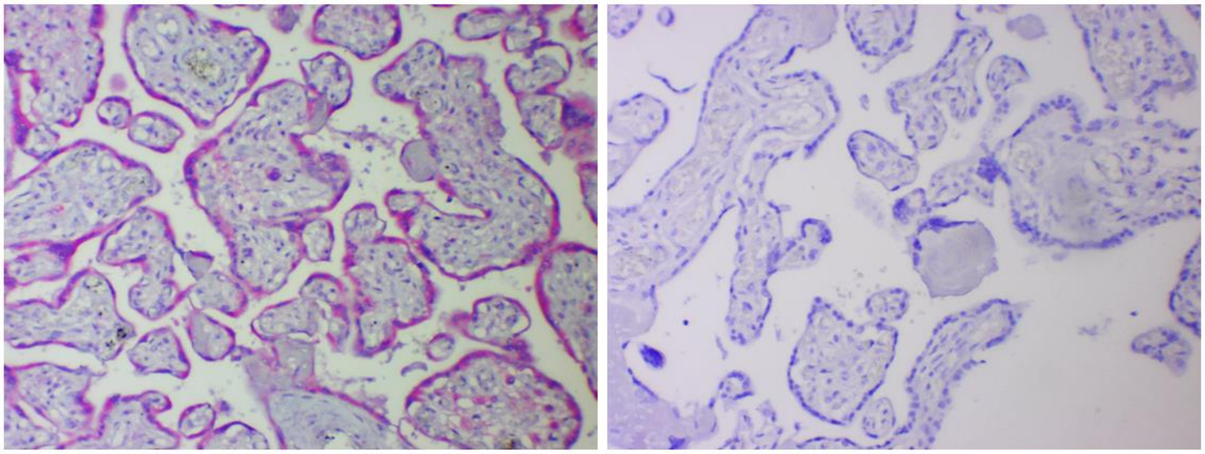

Supplementary Figure S3: Immunohistochemical staining of SVEP1 protein in human placenta. Specific SVEP1 staining is shown (pink, left), which was not observed in tissue sections processed without the primary antibody (right).
